# Cone photoreceptor preservation with laser photobiomodulation in murine and human retinal dystrophy

**DOI:** 10.1002/ctm2.673

**Published:** 2022-02-20

**Authors:** Robert J. Casson, John P. M. Wood, Jack Ao, Jagjit S. Gilhotra, Shane R. Durkin, James Muecke, WengOnn Chan, Glyn Chidlow

**Affiliations:** ^1^ Ophthalmic Research Laboratories University of Adelaide Adelaide South Australia Australia; ^2^ Department of Ophthalmology Royal Adelaide Hospital Adelaide South Australia Australia

Dear Editor,

We used a novel slit lamp‐delivered photobiomodulation (PBM) retinal laser to preserve cone photoreceptors in an animal model of retinitis pigmentosa (RP) and to improve visual acuity in individuals with advanced RP.

RP refers to a genetically heterogenous group of blinding inherited retinal dystrophies (IRDs), and PBM refers to the treatment of tissue with light in the far red to near‐infrared spectrum. Bioenergetic dysfunction and oxidative stress are implicated in the pathogenesis of secondary cone degeneration in RP;^1^, ^2^ and are mitigated by the photonic action of PBM on cytochrome *c* oxidase in the electron transport chain.^3^ To date, PBM research on the retina has almost invariably used light‐emitting diode (LED) systems; however, this methodology suffers the disadvantage that the energy delivery at the level of the retina is uncontrolled. Our experimental 670 nm slit lamp‐delivered retinal laser enables controlled delivery of a known intensity (irradiance) to the retina. The methods are described in the Supporting Information.

We firstly assessed the effect of PBM on a mixed retinal cell culture preparation (including tau‐immunoreactive neurons, rhodopsin‐immunoreactive rods and S opsin‐expressing S‐cones), under conditions of oxidative stress and mitochondrial compromise.^4^, ^5^ Treatment with PBM alone did not detrimentally affect cells at exposures up to 100 mW/cm^2^ (Figure S1). Exposure to either stressor for 24 h resulted in dramatic reduction of rods, which was significantly mitigated by pre‐treatment with PBM (100 mW/cm^2^; Figure 1A, B). Similar results were found with S‐cones (Figure 1C, D) and neurons (Figure S2A). Subsequent investigations using MitoSOX Red and cytochrome oxidase enzyme histochemistry confirmed that PBM treatment (100 mW/cm^2^) also stimulated an immediate, robust, short‐lived increase mitochondrial activity (Figure S2B, F). Finally, using qPCR and western blotting, we showed the elevated expression of photoreceptor genes as well as antioxidant genes in PBM‐treated samples (Figure S2C). We also found that the striking induction in haemoxygenase‐1 evoked by oxidative stress injury was mitigated by PBM treatment (Figure S2D). These data demonstrate that PBM influences mitochondrial function.

**FIGURE 1:**
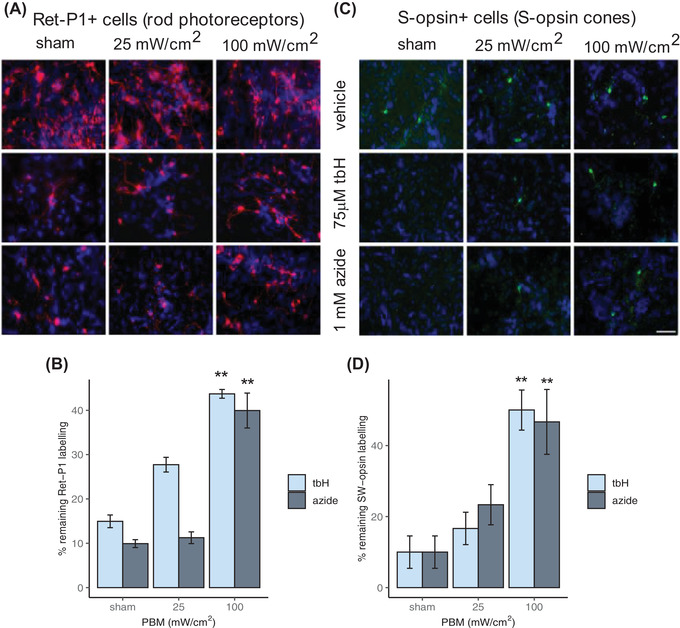
Protection of photoreceptor cells in mixed retinal cultures by PBM. Mixed cultures of retinal cells, consisting of neurons, glia and photoreceptors, were exposed in vitro to PBM at either 25 or 100 mW/cm^2^, or to sham treatment. After 6 h, they were subjected to either oxidative stress (75 μM tert‐butyl‐hydroperoxide; tbH) or mitochondrial compromise (1 mM sodium azide) for a further 24 h. Immunocytochemical labelling for rod photoreceptors (A, B) and for S‐cones (C, D) was significantly decreased by exposure of cultures to both tbH and azide. Pre‐treatment with PBM at 100 mW/cm^2^, but not at 25 mW/cm^2^, significantly alleviated the effects of both toxins for each cell‐type compared to sham. ^*^ *p* < .05, ^**^ *p *< .01, by post hoc Dunnett's test compared to sham group; *n* = 6 determinations (each ‘*n*’ reflects the average of individual values determined from four randomly selected central regions, per coverslip; these data were collated from six separate cultures) for each test group; error bars depict SEM. Scale bar: 50 μm

We then progressed to in vivo investigation of the effects of PBM on retinal cones in *rd1* mice.

We assessed the effect of PBM on cone preservation at P60, using cone cell immunostaining density averaged across each retinal flatmount as the primary outcome. Mice received twice weekly PBM treatment to one eye commencing at P21. At P60, M/L cone density was significantly greater in *rd1* mice treated with PBM at either 25 or 100 mW/cm^2^ compared to shams (Figure 2A). S‐cone cell body density was similarly preserved (Figure 2A). We also assessed the survival of M/L cone outer segments, whose presence is required for detecting light. M/L cone outer segment survival was significantly prolonged by PBM (Figure 2A and Figure S3). Electroretinographic activity in the *rd1* mouse is unrecordable by P30 due to the early onset of cone segment degeneration. Hence, we assessed residual vision by recording the optokinetic reflex at P35 and observed partial preservation of this visual reflex in the PBM‐treated groups (Figure 2A).

**FIGURE 2:**
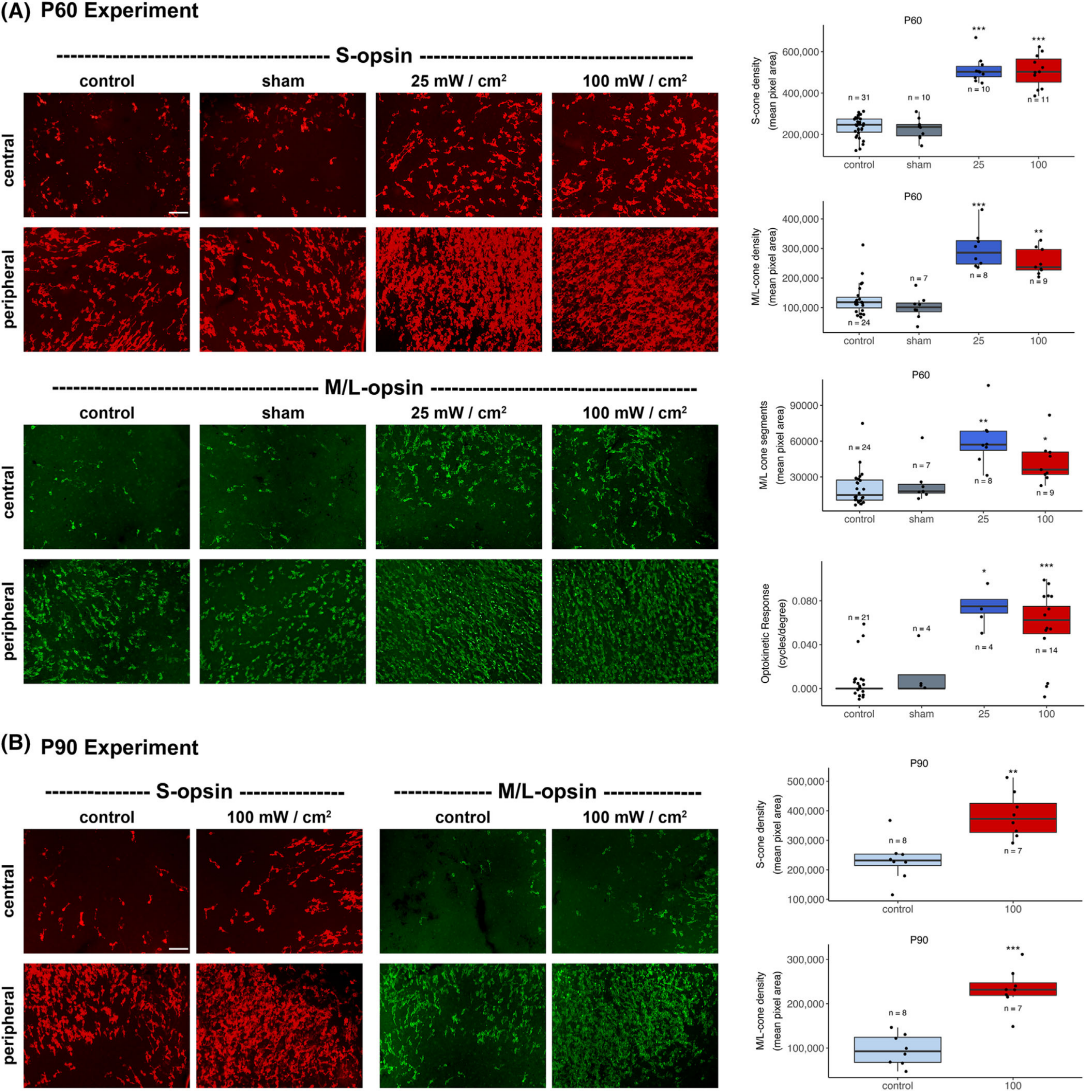
Effect of PBM on S‐opsin^+^ and M/L‐opsin^+^ cone survival in rd1 retinas at P60 and P90. (A) Representative photomicrographs of S‐opsin^+^ and M/L‐opsin^+^ immunoreactivities in *rd1* retinal wholemounts at P60 from the central and peripheral retina in control, sham, 25 mW/cm^2^ PBM and 100 mW/cm^2^ PBM groups. Scale bar: 100 μm. S‐opsin^+^ cone densities, as well as M/L‐opsin^+^ cone densities and cone outer segments, were significantly preserved by treatment with PBM at irradiances of either 25 or 100 mW/cm^2^ as compared to untreated control and sham‐treated mice. The optokinetic reflex was also significantly preserved, indicating that *rd1* mice treated with PBM retained some functional vision. (B) Representative photomicrographs of S‐opsin^+^ and M/L‐opsin^+^ immunoreactivities in *rd1* retinal wholemounts at P90 from the central and peripheral retina in control and 100 mW/cm^2^ PBM groups. Scale bar: 100 μm. Both M/L‐opsin^+^ and S‐opsin^+^ cones were preserved at P90 (E, F). All data represent mean ± SEM. Box length = interquartile range (IQR). Black horizontal line = median, whiskers = 1.5 × IQR; black circles = data points. *** *p* < .001; ** *p* < .01, * *p* < .05, by post‐hoc Dunnett's multiple comparison test versus control group (P60) and Student's paired *t*‐test (P90)

To determine whether the neuroprotective influence of PBM extended to longer durations, we then investigated cone survival at P90. Given the similarity of the effect at both 25 and 100 mW/cm^2^, to reduce animal numbers, we elected to treat at 100 mW/cm^2^W only. We again observed marked preservation of cones in PBM‐irradiated eyes compared to untreated controls (Figure 2B).

Motivated by the safety and efficacy in vivo, we rapidly translated this technology to a phase I trial of patients with advanced RP who had progressed to tunnel vision and impairment of cone‐derived visual acuity: 12 patients were entered into the study and designated as Group 1 (receiving 25 mW/cm^2^) or Group 2 (receiving 100 mW/cm^2^) according to enrolment chronology (Figure S4).

The procedure was well tolerated by all participants and there were no adverse reactions at either irradiance. There were no missing data. Combining groups, patients recovered a mean of 5.4 letters (SD 5.5) at the 8‐week time point (4 weeks after completion of treatment, Figure 3A). The group receiving 100 mW/cm^2^ displayed a greater variance (Figure 3C). Cone‐derived photopic flicker responses were almost completely abolished in all participants with a large within‐ subject and between‐subject variance (Figure 3B). No significant change in electroretinogram amplitude was observed (Figure 3D, F), but responses were essentially unrecordable in these patients due to advanced disease. The procedure had no significant effect on any of the measured ophthalmic parameters. There was a suggestion that the effect on visual acuity was tapering by 6 months (Figure 3E).

**FIGURE 3:**
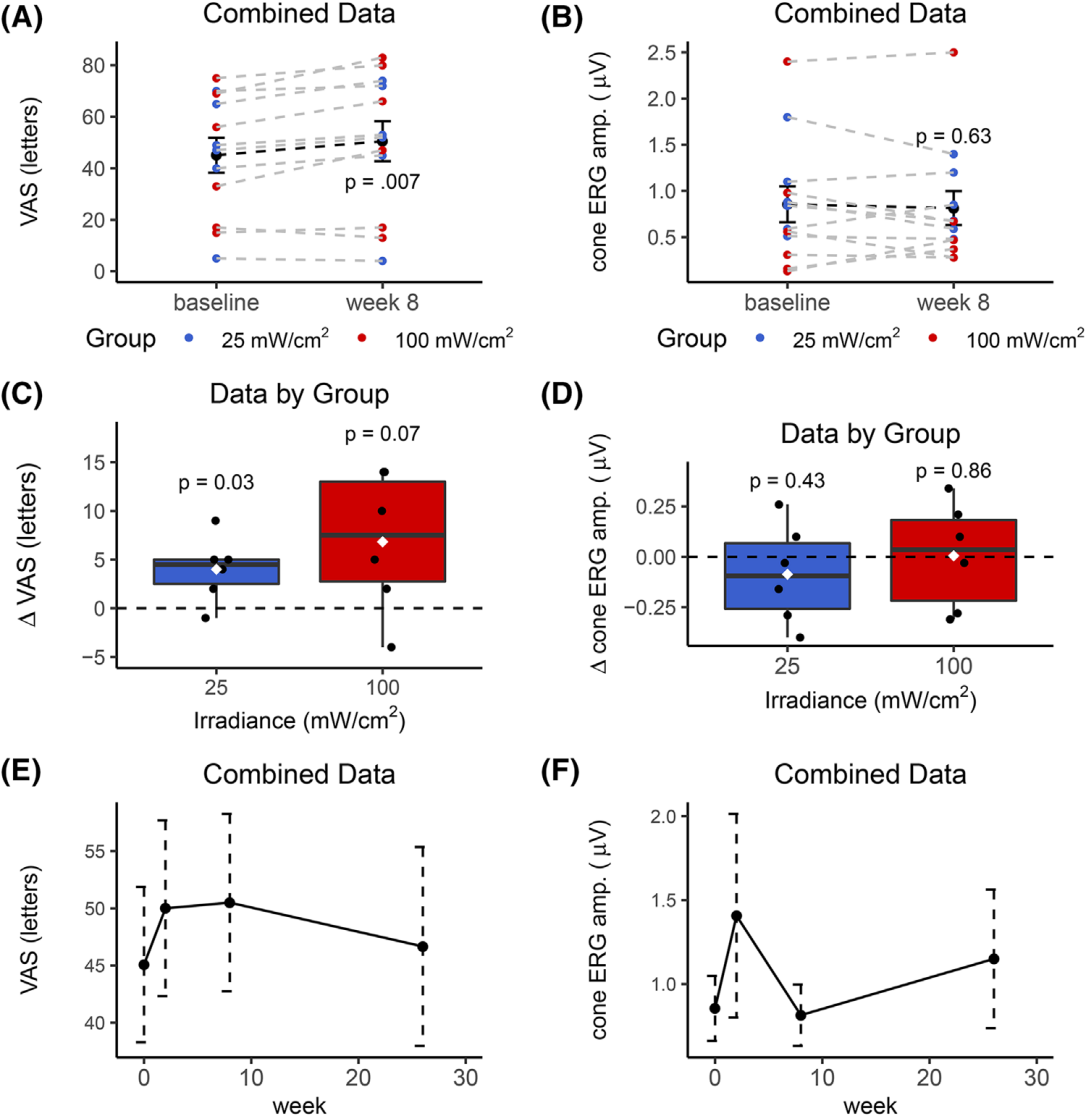
Effect of PBM on visual acuity and electroretinographic (ERG) photopic flicker amplitude (amp.) in individuals with RP. (A) Combining data from both groups, PBM significantly recovered the visual acuity score (VAS) by a mean of five letters at the pre‐defined time point, 4 weeks after the final treatment (week 8; *n *= 12). (C, D) VAS and ERG data by irradiance group; *n* = 6 per group with *p* values comparing week 8 to baseline (dashed horizontal black line at *y* = 0). (E, F) Combined data (*n* = 12) showing change in VAS and the photopic flicker amp. from baseline to the 26‐week endpoint. Error bars depict standard errors. Box length = interquartile range (IQR). Black horizontal line = median, white diamond = mean, whiskers = 1.5 × IQR; black circles = individual data points. *p* Values calculated using Welch's *t*‐test (VAS comparisons) or Wilcoxon test (ERG data) on paired data

PBM is an attractive therapeutic modality for retinal diseases that comprise bioenergetic failure, oxidative stress and/or gliosis components.^6^ In 2013, Kirk et al. reported that 670 nm LED light (Quantum Devices, Barneveld, WI) attenuated the loss of retinal function and structure in a rat model of RP (the P23H rat).^7^ Although cones were not specifically investigated in this study, the results are consistent with the findings from the current study.

The IRDs remain a significant visual health problem and have traditionally been recalcitrant to therapeutic intervention. However, based on the results of improved multi‐luminance mobility testing in a phase III randomized controlled trial (RCT),^8^ the Food and Drug Administration recently approved voretigene neparvovec‐rzyl for the treatment of patients with biallelic *RPE65‐*mediated IRD.^9^ RPE65 mutations account for approximately .1–1% of IRD. Gene therapy for other recessive IRDs is an explosive research area. However, these genetic engineering techniques are disease specific, expensive and restricted to high socio‐economic index populations. The development of artificial retinal implants is largely targeted at IRDs but has had limited clinical impact to date. Recently, Campochiaro et al. reported that oral N‐acetyl‐cysteine preserved 2–3 letters in each of the three cohorts of 10 patients receiving different doses of N‐acetyl‐cysteine over a 24‐week period.^10^ Hence, the five‐letter short‐term improvement noted in the current study compares favourably with oral anti‐oxidants, but it must be noted that this is within the range of the test‐retest variability.

A relatively low‐cost strategy that preserves central vision irrespective of the rod gene defect would be a major medical breakthrough at the individual and community level. Our translational research strongly motivates further studies investigating PBM as a treatment for RP. Further work is required to determine the optimal frequency of delivery and to assess the effect in a suitably powered RCT.

## CONFLICT OF INTEREST DISCLOSURES

The authors have declared that no conflict of interest exists.

## Supporting information

Supporting Information
