# Supplementary material for: Cone photoreceptor preservation with laser photobiomodulation in murine and human retinal dystrophy
Source: Clin Transl Med. 2022 Feb 20;12(2):e673. doi: 10.1002/ctm2.673 (PMC8858622; doi:10.1002/ctm2.673)
Supplement: Supplementary file 1 — Supporting Information [file CTM2-12-e673-s001.docx]

**Supplementary Material**

# **Methods**

# Photobiomodulation Retinal Laser

The PBM laser system comprised a custom-designed, slit lamp microscope-mounted Integre near-infrared laser (Ellex Medical Lasers, Adelaide, South Australia, Australia) incorporating a 670-nm light source emitting a beam 4.5 mm in diameter with a flat-top profile.

# Clinical Trial

## Clinical Trial Primary Outcome Measure

We conducted a phase 1 single ascending dose study (ACTRN12618000651280), assessing the safety of PBM to the macula in individuals with RP. The Transparent Reporting of Evaluations with Nonrandomized Designs guidelines were followed.^1^ The primary outcome measure was the safety profile, and the secondary outcomes were the change in visual acuity and photopic flicker ERG. The target population was individuals with advanced RP and partial loss of central vision.

## Participants and Protocol

We aimed to include a total of 12 patients in the clinical trial. Our protocol designated 6 patients to receive the lower PBM irradiance setting (25 mW/cm^2^; Group 1) and, upon completion of the treatment and safety determined after 6 months follow-up, a further 6 patients would be designated to the higher PBM irradiance setting (100 mW/cm^2^; Group 2).

A participant flow chart is shown in Supplementary Fig. 1. Irradiance parameters were selected based on our previous animal safety studies and on settings used in our previous clinical trial assessing the effect of laser PBM on DME.^2^

A total of 25 patients from RJCs, JSGs and SRDs private clinical practices and the Retinal clinics at the Royal Adelaide Hospital were identified as potential participants; 12 declined to participate and 13 were screened. One patient failed screening because the visual acuity was still within normal limits (20/30 in the worst eye).

The data collection and laser treatment were conducted in the Ophthalmology Department at the RAH. Patients had to be ≥ 18 years of age and have a best-corrected visual acuity (BCVA) between 20/40 and 20/800 (5 – 70 letters) with visual impairment attributed to RP. Patients were excluded if they had had an intraocular procedure or laser treatment within the previous 6 months, or if they had cystoid macula edema. Patients underwent a baseline assessment (week 0) then received 2 treatments per week (separated by approximately 48 hours) for 4 weeks. Patients were encouraged to attend all visits and were given verbal and written reminders of ongoing appointments. At baseline, the best-corrected logarithm of the minimum angle of resolution visual acuity was recorded using an Early Treatment Diabetic Retinopathy (ETDRS) chart at 4 m (or 1 m if the BCVA was < 20/200) Slit lamp examination of the anterior segment was performed by an experience ophthalmologist (RJC) and intraocular pressure measured with Goldmann tonometry. The pupil was dilated with tropicamide 0.5% and the fundus examined. Spectral domain optical coherence tomography of the macula using the Cirrus™ HD-OCT (Carl Zeiss Meditec AG, Dublin CA), fundus photography and photopic flicker ERG with the RETeval^®^ system (LKC Technologies, Gaithersburg, MD) were performed. Baseline assessments were repeated after 2, 8 and 24 weeks. The visual acuity was measured by a single experienced ophthalmic nurse and the patient was encouraged to read as many letters on the ETDRS chart as possible at each visit. As per the ETDRS chart protocol, the examiner stopped the test only when it became evident that no further meaningful readings were being made, despite urging the subject to guess.

The development of any ocular adverse reaction and/or reduction in visual acuity of ≥ 5 letters at the 2-week visit mandated withdrawal. Any ocular adverse effects in Group I prohibited proceeding to Group II. Any undesirable clinical occurrence in a patient whether it was considered to be device related or not, that included a clinical sign, symptom or condition and/or an observation of an unintended technical performance or performance outcome of the device were carefully recorded.

## Treatment

The pupil was dilated with tropicamide 0.5% and the cornea anesthetized with topical amethocaine. At the slit lamp, a Mainster focal/grid lens was used to visualize the fundus. Each treatment consisted of a 90 s exposure of the macular region to the 4.5 mm diameter laser beam. The laser intensity was set at 25mW/cm^2^ for the low dose (Group I) patients and 100 mW/cm^2^ for high dose (Group II) patients.

## Study approval

The experiments involving animals adhered to the Australian code for the care and use of animals for scientific purposes (the Code) and were approved by the University of Adelaide Animal Ethics Committee. The clinical study was conducted in accordance with the Declaration of Helsinki and was approved by the Central Adelaide Local Health Network Human Research Ethics Committee. Written informed consent was received from the participants prior to inclusion in the study.

# Cell Culture Studies

Cultures were prepared by enzymatic and mechanical dissociation of newborn Sprague-Dawley rat pups, as previously described.^3^ Cultures comprised the majority of cell-types present in the newborn rat retina, including neurons, S-cones and rhodopsin-labelling cells, designated as rod photoreceptors. Cells were seeded onto borosilicate glass coverslips pre-coated with 10 µg/ml poly-L-lysine and maintained in standard Minimal Essential Medium (MEM) containing phenol red indicator, supplemented with 10 % (v/v) fetal bovine serum and antibiotics for 7-8 days.

For laser treatment, coverslips with adherent cells were placed into culture plates containing serum-free MEM lacking phenol red, and positioned on a pre-warmed, bespoke, horizontal platform attached to the chin rest of the slit lamp delivery system. Treatments were applied centrally to individual coverslips at a range of radiant exposures (25, 100, 250, 450 mW/cm^2^) for 90 s. The area covered by PBM treatment was of 15.9 mm^2^ (4.5 mm diameter). Sham treatment was carried out in the same manner as for PBM application except that exposure was only to the laser aiming beam for the allotted time and not the laser. After treatment, cells were replaced into standard medium.

In some experiments, 6 h after PBM exposure, one of two stressors was added: 75 µM tert-butyl hydroperoxide, to induce oxidative stress, or 1 mM sodium azide, to compromise mitochondrial function. Stressors were left for a further 24 hours before cultures were fixed with neutral-buffered formalin (15 minutes) and processed for immunocytochemistry as described previously.^3^ Cultures were immunocytochemically labelled as follows: neurons for tau (Dako, Denmark; 1:5000), rod photoreceptors for rhodopsin (clone Ret-P1; Santa Cruz Biotechnology In., Santa Cruz, USA; 1:5000) and SW cones for S-opsin (sc-14363, Santa Cruz Biotechnology Inc., Santa Cruz, USA; 1:1000). Nuclear counter-staining of cells was achieved with a final five-minute incubation of coverslips in 500 ng/ml 4′,6-diamidino-2-phenylindole. Images were collected from each of 6-8 different cultures for each treatment and Image-J software (NIH, Bethesda, Maryland, USA) subsequently used to quantify labelling for each specific cell-type being investigated. Data were compared using one-way analysis of variance followed by Tukey’s test for multiple comparisons; significance was denoted by P < 0.05. The dosimetry response of retinal neurons and photoreceptors in culture to PBM is shown in Supplementary Figure 1.

In order to study the effects of laser-delivered PBM on mitochondrial respiration, we conducted live cell imaging after incubating treated cultures with 5 µmol/l MitoSOX Red dye (ThermoFisher Scientific, Scoresby, Victoria, Australia) for 10 min. This dye is oxidised by superoxide production in the mitochondria of live cells to form a product which is highly fluorescent upon binding to nucleic acids. Cells were co-labelled with the live cell nuclear-binding dye, Hoechst 33342.

In order to study the effects of laser-delivered PBM on mitochondrial cytochrome C oxidase (COX IV) activity in situ, retinal cultures were exposed to PBM and then immediately subjected to mitochondrial compromise sodium azide (1 mM) for 2 hours prior to conducting COX IV enzyme histochemistry. For the assay, cultures on coverslips were rinsed in PBS, immediately stored at -80ºC for 15 min to aid permeabilisation, thawed, and then incubated for 1 hour at 37ºC in a reaction medium containing 2.5 mg 3,3’-diaminobenzidine, 5 mg of cytochrome c, 90μg catalase, and 4% sucrose in 5 mL 0.05M phosphate buffer (pH 7.4). To terminate each reaction, coverslips were rinsed in distilled water, fixed for 5 minutes in neutral buffered formalin, rinsed, dehydrated, and then mounted.

## Real-time RT-PCR

Reverse transcription polymerase chain reaction (RT-PCR) studies were carried out as described previously ^4,5^. In brief, total RNA was isolated from retinal cultures in individual well plates. First strand cDNA was then synthesised from DNase-treated RNA. Real-time PCR reactions were carried out in 96-well optical reaction plates using the cDNA equivalent of 10 ng total RNA for each sample in a total volume of 20 μl containing 1×SYBR Green PCR master mix (BioRad, Gladesville, Australia) forward and reverse primers. The thermal cycling conditions were 95°C for 3 min and 40 cycles of amplification comprising 95°C for 12 s, annealing temperature (Supplementary Table 1) for 30 s and 72°C for 30 s. After the final cycle of the PCR, primer specificity was checked by the dissociation (melting) curve method. PCR assays were performed using the CFX cycler (Bio-Rad) and all samples were run in duplicate. Threshold cycles were calculated using CFX Manager Software (Bio-Rad). All values were normalised using the endogenous reference gene hypoxanthine phosphoribosyltransferase 1 (HPRT1) and results expressed as mean ± SEM. Primer pairs (Supplementary Table 1) were designed from sequences contained in the Genbank database using the primer design software Primer 3 (<http://bioinfo.ut.ee/primer3-0.4.0/primer3/>) and were selected to amplify sequences that spanned at least one intron. Primer sequences were analyzed for T_m_ (melting temperature), secondary structure and primer-dimer formation with NetPrimer analysis software ([http://www.premierbiosoft.com/netprimer](http://www.pubmedcentral.nih.gov/redirect3.cgi?&&reftype=extlink&artid=525120&iid=17437&jid=83&FROM=Article|Body&TO=External|Link|URL&article-id=525120&journal-id=83&&http://www.premierbiosoft.com/netprimer)) and verified for their specificity to the target sequence. The results showed that all mRNAs were amplified with high efficiency and linearity during real-time PCR. Mean amplification efficiencies, as determined by plotting cycle threshold as a function of initial cDNA quantity, ranged from 1.9 – 2.0. Results obtained were, therefore, quantified using the comparative threshold cycle (C_T_) method (ΔΔC_T_) for relative quantitation of gene expression, with a minor correction for amplification efficiency^6^.

## Western blotting

Tissues were processed for Western blotting as previously described ^4^. In brief, retinal cultures in individual 6-well plates were extracted and sonicated in homogenization buffer (20mM Tris-HCl, pH 7.4, 25^o^C; containing 2mM EDTA, 0.5mM EGTA, 1mM dithiothreitol, 50μg/ml leupeptin, 50μg/ml pepstatin A, 50μg/ml aprotinin and 0.1mM phenylmethylsulphonyl fluoride). An equal volume of sample buffer (62.5mM Tris-HCl, pH 7.4, containing 4% SDS, 10% glycerol, 10% β-mercaptoethanol and 0.002% bromophenol blue) was then added and samples were boiled. Electrophoresis was performed on 10% denaturing polyacrylamide gels after which proteins were transferred to polyvinylidine fluoride membranes for immunoprobing. Membranes were incubated with anti-HO-1 antibody (1:2000, cat#SPA-895, Enzo Life Sciences) or anti-SOD-1 antibody (1:1000, cat#574597, Calbiochem) or anti-actin antibody (1:5000, clone AC-15, Sigma) and labeling carried out using a multi-step detection procedure: first, appropriate biotinylated secondary antibodies were reacted with membranes and then streptavidin-peroxidase conjugates were applied. Blots were developed with a 0.016% solution of 3-amino-9-ethylcarbazole in 50 mM sodium acetate (pH 5) containing 0.05% Tween-20 and 0.03% H_2_O_2_. Images were acquired from labelled blots using a Canon CanoLide flatbed scanner and analysed for densitometry using the software program, Adobe PhotoShop CS2. Densitometry values were then normalised for β-actin.

# Animal Studies

Mice were divided into three treatment groups: sham (aiming beam only), lower dose PBM laser (25 mW/cm^2^) and higher dose PBM laser (100 mW/cm^2^). Since the photoreceptor loss occurs bilaterally, one eye received PBM laser treatment while the fellow untouched eye served as a paired control. Treatment began from P21 and occurred twice weekly until euthanasia.

Topical oxybuprocaine was applied to the ocular surface and the pupil dilated with tropicamide. Mice received inhalational anesthesia with isoflurane delivered via a nosecone throughout the procedure. A coverslip was placed on the cornea and mice were positioned on a custom-designed platform at the slit lamp laser delivery system. The laser was centered on the optic nerve and the fundus was exposed to PBM laser for 90 s.

## Optokinetic Response

The optokinetic reflex is commonly used to measure the visual function of an animal by observing the maximum spatial frequency of a rotating visual stimulus for which the animal responds with a head reflex. Animals were placed on a platform on a floor mirror surrounded by computer monitors that formed an enclosed area. Vertical sine wave gratings (100% contrast) were projected on the computer monitors. The spatial frequencies tested were 0.05, 0.075, 0.1, 0.2, 0.3, 0.4, 0.5, and 0.6 cycles per degree. A camera was placed above the platform to observe and record the animal’s head movements. Mice were placed one at a time on the platform and allowed to acclimatize to their surroundings before starting the stimulus. The stimulus consisted of a grating perceptible to the mouse that was projected on the cylinder wall which rotated at a constant 12 degrees/s. Two independent experimenters masked to the treatment groups monitored the head reflex characterized by the animal displaying reflexive head movements corresponding to the direction and speed of the cylinder rotation that was not accompanied by any other body movements. A positive response was recorded only if the reflexive head movement occurred within the first 15 s of the stimulus and there was agreement between the experimenters. Assessment of the left or right eye was dependent on cylinder direction. Clockwise direction of the cylinder corresponded to the left eye whilst counter-clockwise direction was related to the right eye. The spatial frequency of the grating was progressively increased until the animal no longer responded. The maximum spatial frequency for a positive head reflex was recorded. This was repeated for each eye.

## Tissue processing and immunohistochemistry

All mice were euthanized by transcardial perfusion with physiological saline under terminal anaesthesia (100 mg/kg body weight ketamine and 10 mg/kg body weight xylazine) followed by decapitation. The superior aspect of each cornea was marked before globes were enucleated. For wholemount double labeling immunohistochemistry, eyes were fixed in 4% (w/v) neutral buffered formalin for 24 h and dissected into posterior eye-cups. The corneal mark was used to orient the eye and a small radial cut was made in the superior retina while it was still attached to the retinal pigment epithelium in the eye-cup. Retinas were removed and prepared as flattened wholemounts by making another four radial cuts. Retinal wholemounts were then stored in PBS, prior to incubating in PBS containing 1% (v/v) Triton X-100 detergent (T) for 1 h at room temperature. Next, retinas were incubated in PBS-T containing 3% (v/v) normal horse serum (NHS)-T for 1 h at room temperature to block non-specific antibody binding. Retinas were then incubated overnight at 4 °C with a combination of primary antibodies diluted in NHS-T. OPN1SW antibody (1:1500, sc-14363, Santa-Cruz) was used to detect S-cones, whilst anti-R/G opsin antibody (1:1500, AB5405, Merck-Millipore) was used to detect M/L- cones. On day 2, retinas were washed for 1 h at room temperature in PBS-T, then incubated overnight at 4 °C with a combination of AlexaFluor-488 and -594 conjugated secondary antibodies (1:250; Invitrogen, Carlsbad, CA, USA) diluted in NHS-T. Finally, retinas were washed in PBS for 1 h at room temperature prior to mounting with the photoreceptor side facing up, using anti-fade mounting medium (Dako, Santa Clara, CA, USA).

## Image acquisition and quantification

All analyses were conducted in a masked fashion. Photomicrographs of wholemounts were taken with an epifluorescent microscope with attached fluorescent optics (BX-61; Olympus, Mount Waverly, VIC, Australia). Rectangular areas of 526.5 x 422.5 µm were photographed adjacent to the optic disk and 2 mm away from the optic disk in each of the retinal quadrants. This yielded 8 images per retina (4 central and 4 peripheral). Quantification of cone survival was performed using Image-J software (NIH, Bethesda, Maryland, USA). Initially, however, images were processed in Photoshop CS3 (Adobe). Images were corrected for uneven lighting using a flatten filter and where necessary linear gradient tool, then sharpened, levels enhanced, and finally converted to 8-bit mode.

Cone cell bodies were identified by their wide ovoid morphology and segments by their narrow, bundle-like appearance. For determination of total S-cone and total M/L-cone labelling, images were manually thresholded until all cone cell bodies and segments were highlighted. The area in pixels was then calculated with the “analyze particles” function, using a minimum size of 5 square pixels. As shown in supplementary Figure 4, ML-cone segments stained with greater fluorescent intensity then cell bodies. This permitted quantification of M/L-cone segment survival (which necessarily comprises both genuine M/L-cones and dual cones). Quantification of segments was performed by adjusting the image threshold to isolate the more intensely stained M/L-cone segments. The area in pixels was then calculated with the “analyse particles” function, using a minimum size of 5 square pixels. In contrast to M/L-cones, S-cone cell bodies and segments stained with similar intensity. Separate quantification of S-cone segments from cell bodies could not be accurately attained with image threshold adjustment. Representative images of M/L-opsin^+^ and S-opsin^+^ immunoreactivities (IR) in retinal wholemounts from *rd1* mice are shown in Supplementary Figure 4.

# Statistics

Initial exploratory analyses were performed on all data including construction of box plots and examination of the distribution and heterogeneity of residuals with histograms and Q-Q plots. Where normality was met, comparisons between groups were made with either a post hoc Dunnett’s test or Welch’s paired *t*-test. If normality was not met a non-parametric test was used. A p value < 0.05 was considered statistically significant. Statistical analyses were performed using the R statistical software.^7^

# References

1. Des Jarlais DC, Lyles C, Crepaz N, Group T. Improving the reporting quality of nonrandomized evaluations of behavioral and public health interventions: the TREND statement. *Am J Public Health.* 2004;94(3):361-366.

2. Shen W, Teo KYC, Wood JPM, et al. Preclinical and clinical studies of photobiomodulation therapy for macular oedema. *Diabetologia.* 2020.

3. Wood JP, Mammone T, Chidlow G, Greenwell T, Casson RJ. Mitochondrial inhibition in rat retinal cell cultures as a model of metabolic compromise: mechanisms of injury and neuroprotection. *Invest. Ophthalmol. Vis. Sci.* 2012;53(8):4897-4909.

4. Chidlow G, Holman MC, Wood JP, Casson RJ. Spatiotemporal characterization of optic nerve degeneration after chronic hypoperfusion in the rat. *Invest. Ophthalmol. Vis. Sci.* 2010;51(3):1483-1497.

5. Chidlow G, Wood JP, Manavis J, Osborne NN, Casson RJ. Expression of osteopontin in the rat retina: effects of excitotoxic and ischemic injuries. *Invest. Ophthalmol. Vis. Sci.* 2008;49:762-771.

6. Pfaffl MW. A new mathematical model for relative quantification in real-time RT-PCR. *Nucleic Acids Res.* 2001;29(9):e45.

7. R Core Team. A language and environment for statistical computing. R Foundation for Statistical Computing, Vienna, Austria. . [*https://www.R-project.org/*](https://www.R-project.org/)*.* 2020.

Supplementary Table 1. Primer sequences for mRNAs amplified by qPCR

| **mRNA** | **Primer sequences** | **Product** | **annealing temperature** | **Accession**  **number** |
| --- | --- | --- | --- | --- |
| catalase | 5’-GCCAGAAGAGAAACCCACAAA-3’  5’-TCGGTCGCTGAACAAGAAAG-3’ | 110 | 63ºC | NM_012520.2 |
| cone arrestin | 5’-TCATTCGGGAGTTCACAGAGAC-3’  5’-GTTTGCCATCCAGTGCCAG-3’ | 117 | 61º C | NM_001190993.1 |
| GPX-1 | 5’-GGGACTACACCGAAATGAATG-3’  5’-CACCTCGCACTTCTCAAACAA-3’ | 191 | 61°C | NM_030826.4 |
| HPRT1 | 5’-GTCATCAGCGAAAGTGGAAAAG-3’  5’-ATCAAAAGGGACGCAGCAAC-3’ | 206 | 61°C | NM_012583 |
| peroxiredoxin-5 | 5’-AAAGGAGCAGGTTGGGAGTG-3’  5’-GCAGATGGGTCTTGGAACAG-3’ | 217 | 63ºC | NM_053610 |
| rhodopsin | 5’-CTCCATCTACAACCCAATCATC-3’  5’-ACTCCTACAGTCAGCCACAGTC-3’ | 187 | 63ºC | NM_033441 |
| S-opsin | 5’-CCTCTTTCCCTCATCTGCTTCTC-3’  5’-ACCTCCCGTTCAGCCTTTTG-3’ | 107 | 63ºC | NM_031015 |
| GNAT1 | 5’-GACGACGAAGTGAACCGAATG-3’  5’-GTTGAGGAAGAGCACGATGGA-3’ | 99 | 63ºC | NM_001108780.2 |

**
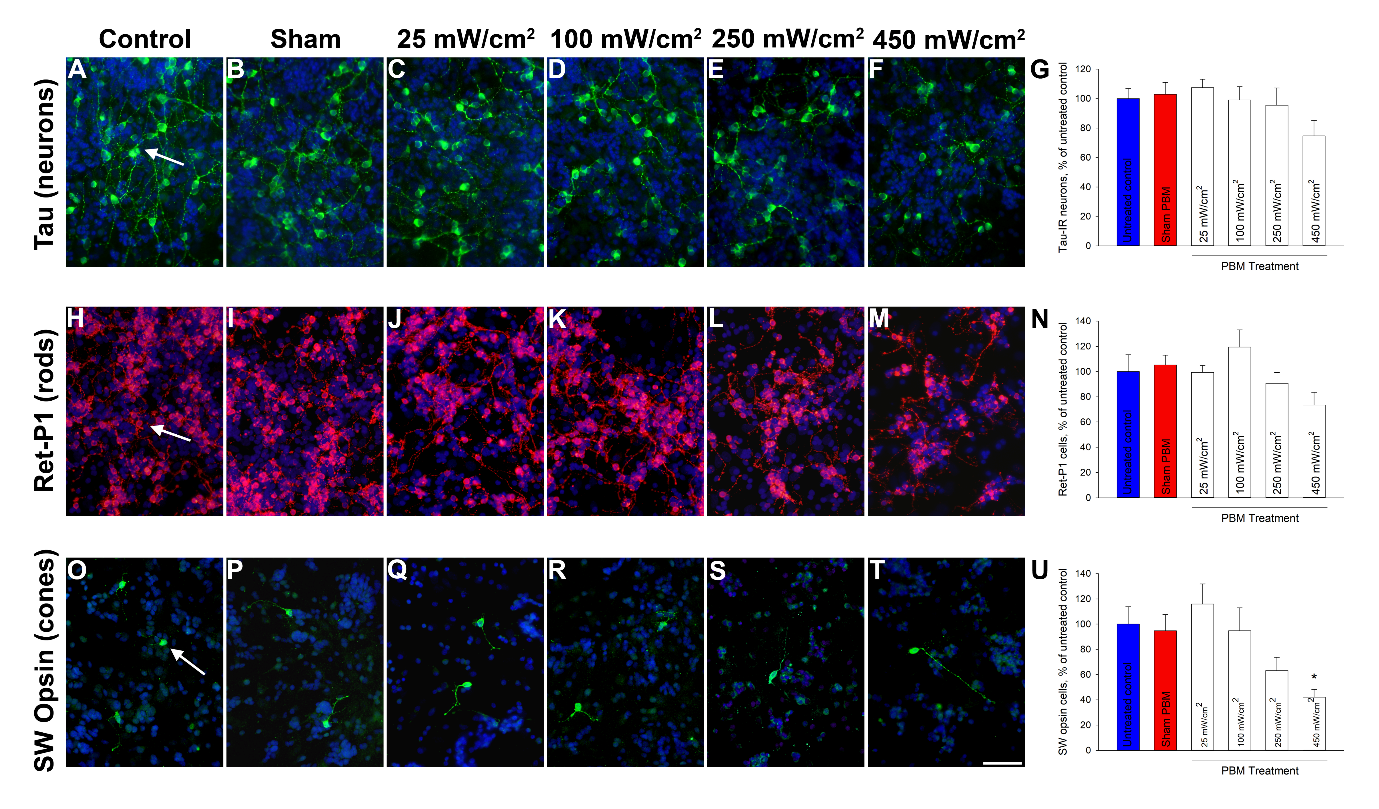
**

Supplementary Figure 1. Dosimetry response of retinal neurons and photoreceptors in culture to PBM. Cultures were exposed to 90 seconds of PBM laser in vitro at a range of energy settings and then fixed after 24 hours to determine potential cytotoxicity. Neither tau-labelled neurons (A-G) nor rhodopsin-labelled rod photoreceptors (H-N) were detrimentally affected by PBM laser application up to 250 mW/cm^2^. At 450 mW/cm^2^, however, there was a tendency for a reduction in labelling for both cell-types, although this effect was not of statistical significance. In the case of SW opsin-labelled cone photoreceptors (O-U), however, there was an apparent reduction when PBM was applied at above 100 mW/cm^2^: this effect was determined to be significant at 450 mW/cm^2^. ^*^*P* < 0.05 by one-way ANOVA plus Dunnett’s test for multiple comparisons; *n*=6 determinations for each test group. Scale bar: 50 µm.


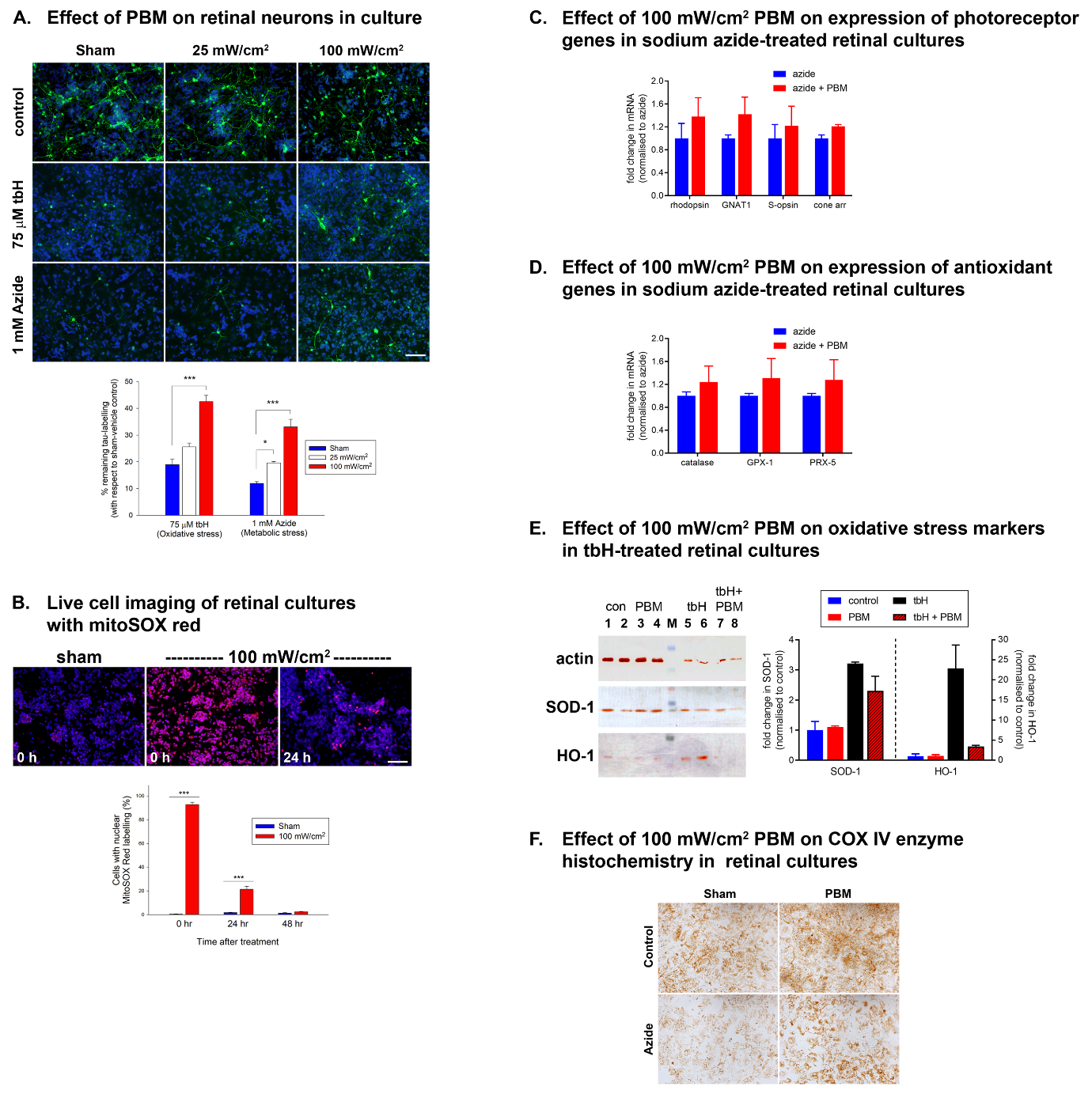


Supplementary Figure 2. Effect of PBM on retinal cultures. (A) Effect of PBM on survival of tau-immunolabelled retinal neurons subjected to oxidative stress (tbH) or metabolic stress (sodium azide). PBM laser application at both 25 and 100 mW/cm^2^ augmented survival of retinal neurons in both injury settings. Scale bar: 50 µm. Data are expressed as mean ± SEM. ****p*<0.001, **p*<0.05 by one-way ANOVA plus Tukey’s post hoc test, where *n*=6 determinations, from separate cultures. (B) Representative live cell imaging after incubating retinal cultures with MitoSOX Red. Increased mitochondrial activity was observed immediately after PBM (0 h) but had returned to baseline by 48 h. Scale bar: 50 µm. Data are expressed as mean ± SEM. ****p*<0.001, by Student’s unpaired t-test (PBM vs sham), where *n*=6 determinations, from separate cultures. (C) Effect of PBM on the levels of rod photoreceptor (rhodopsin and GNAT1) and cone photoreceptor (S-opsin and cone arrestin) mRNAs in retinal cultures subjected to metabolic stress (1 mM sodium azide). Data are normalised to the endogenous gene HRPT-1 and expressed as mean ± SEM, where *n*=3. Relative to the azide-treated group, PBM-treated cultures displayed higher levels of photoreceptor genes. (D) Effect of PBM on the levels of antioxidant (catalase, glutathione peroxidase-1 (GPX-1), peroxiredoxin-5 (PRX5)) mRNAs in retinal cultures subjected to metabolic stress (1 mM sodium azide). Data are normalised to the endogenous gene HRPT-1 and expressed as mean ± SEM, where *n*=3. Relative to the azide-treated group, PBM-treated cultures displayed higher levels of antioxidant genes. (E) Effect of PBM on the levels of hemeoxygenase-1 (HO-1) and superoxide dismutase-1 (SOD-1) proteins in retinal cultures subjected to oxidative stress (75 μM tbH) injury. Data are normalised to the endogenous gene actin and expressed as mean ± SEM, where *n*=3. There was a modest upregulation of SOD-1 and a marked upregulation of HO-1 in the tbH samples compared to control and PBM treated samples. In samples subjected to tbH plus PBM, the oxidative stress-induced upregulation in both proteins was diminished. (F) Effect of PBM on the cytochrome C oxidase (COX IV) enzyme histochemistry in retinal cultures. Photomicrographs are shown of control retinal cultures and retinal cultures subjected to metabolic stress (1 mM sodium azide). There was a modest qualitative increase in COX IV activity in PBM-treated samples.


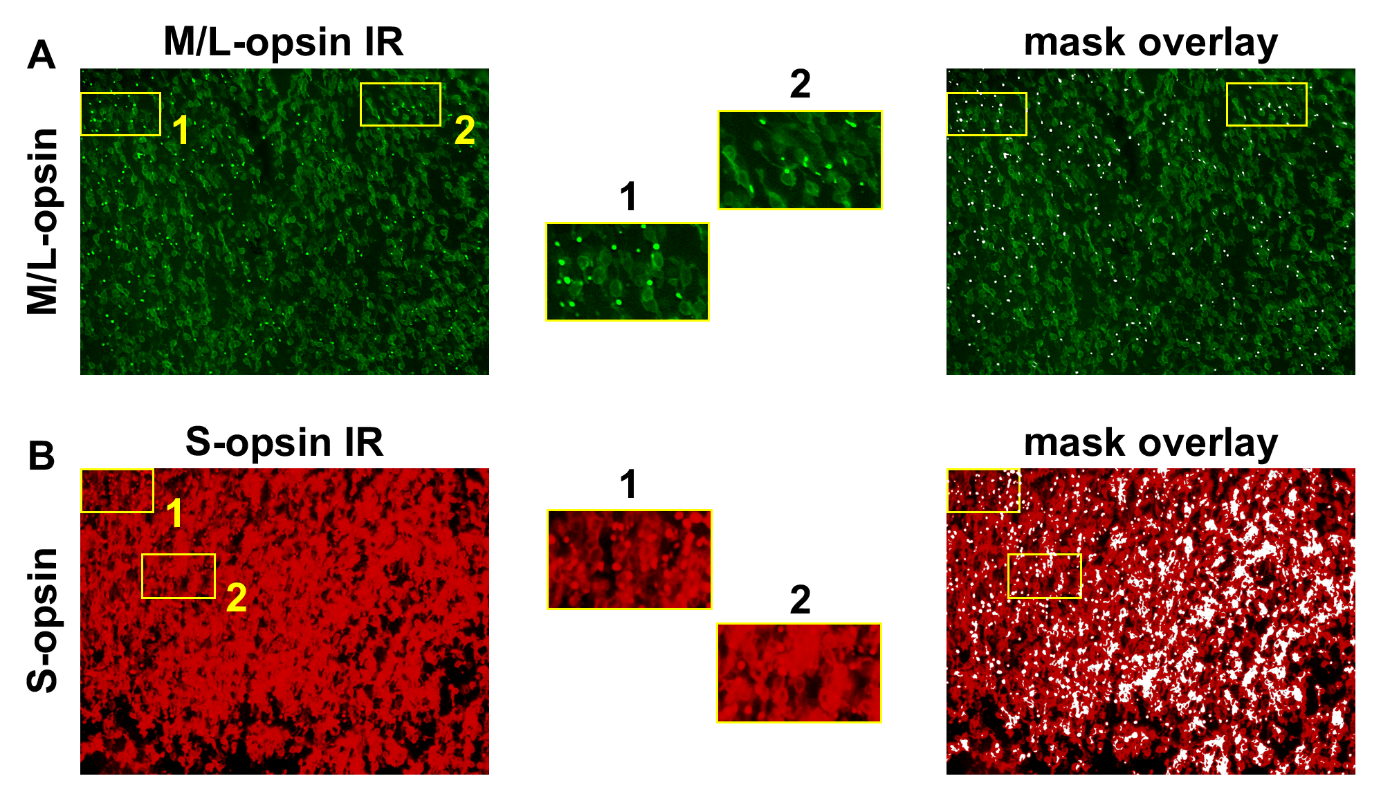


Supplementary Figure 3. Representative images of M/L-opsin^+^ (A) and S-opsin^+^ (B) immunoreactivities (IR) in retinal wholemounts from 100 mW/cm^2^ PBM-treated *rd1* mice at P60. Insets 1 and 2 are magnified views of two regions from each photomicrograph. (A) For M/L-opsin^+^, cell bodies stain lightly, but segments stain intensely (see insets). Thus, segments can be differentiated from cell bodies using image thresholding. The right panel shows the ML-opsin^+^ IR overlaid with the mask derived from image thresholding (white represents areas to be quantified). It can be seen that the mask recapitulates the distribution of immunolabelled segments. (B) For S-opsin^+^, cell bodies and segments both stain intensely (see insets). Thus, segments cannot be routinely differentiated from cell bodies using image thresholding, as illustrated by the S-opsin^+^ IR overlaid with the mask derived from image thresholding. The mask does not recapitulate the distribution of immunolabelled segments. Thus, for quantification of outer segment survival, only ML-opsin^+^ images were used, which represent both genuine ML-opsin^+^ cones and dual cones, but not S-opsin^+^ cones.

Supplementary Figure 4. Participant flow chart.
